# Can motor volition be extracted from the spinal cord?

**DOI:** 10.1186/1743-0003-9-41

**Published:** 2012-06-19

**Authors:** Abhishek Prasad, Mesut Sahin

**Affiliations:** 1 Department of Biomedical Engineering, University of Miami, Miami, FL, USA; 2 Department of Biomedical Engineering, New Jersey Institute of Technology, Newark, NJ, USA

**Keywords:** Brain-computer, Brain-machine, Neural interface, Rubrospinal, Descending tracts

## Abstract

**Background:**

Spinal cord injury (SCI) results in the partial or complete loss of movement and sensation below the level of injury. In individuals with cervical level SCI, there is a great need for voluntary command generation for environmental control, self-mobility, or computer access to improve their independence and quality of life. Brain-computer interfacing is one way of generating these voluntary command signals. As an alternative, this study investigates the feasibility of utilizing descending signals in the dorsolateral spinal cord tracts above the point of injury as a means of generating volitional motor control signals.

**Methods:**

In this work, adult male rats were implanted with a 15-channel microelectrode array (MEA) in the dorsolateral funiculus of the cervical spinal cord to record multi-unit activity from the descending pathways while the animals performed a reach-to-grasp task. Mean signal amplitudes and signal-to-noise ratios during the behavior was monitored and quantified for recording periods up to 3 months post-implant. One-way analysis of variance (ANOVA) and Tukey’s post-hoc analysis was used to investigate signal amplitude stability during the study period. Multiple linear regression was employed to reconstruct the forelimb kinematics, *i.e.* the hand position, elbow angle, and hand velocity from the spinal cord signals.

**Results:**

The percentage of electrodes with stable signal amplitudes (p-value < 0.05) were 50% in R1, 100% in R2, 72% in R3, and 85% in R4. Forelimb kinematics was reconstructed with correlations of R^2^ > 0.7 using tap-delayed principal components of the spinal cord signals.

**Conclusions:**

This study demonstrated that chronic recordings up to 3-months can be made from the descending tracts of the rat spinal cord with relatively small changes in signal characteristics over time and that the forelimb kinematics can be reconstructed with the recorded signals. Multi-unit recording technique may prove to be a viable alternative to single neuron recording methods for reading the information encoded by neuronal populations in the spinal cord.

## Background

There are over 2.5 million people living with spinal cord injury (SCI) worldwide with over 250,000 cases of SCI patients in the USA alone [1]. Depending on the level of injury, these individuals can be either quadriplegic (no sensation in the upper and lower limbs) or paraplegic (no sensation below waist level) [2]. The mean life expectancy of those surviving the initial injury is over 40 years [3,4] as a result of which there are considerable costs associated with primary care and loss of income. Additionally, there are thousands of new patients each year with other neurodegenerative disorders. Neuroprosthetic development has been driven and motivated by the above numbers in the last few decades for improving the quality of daily life for these individuals [5-8]. Current advances in the neuroprosthetic technology have restored communication and control in animal models and human subjects [9,10]. However, the current state of brain-machine interface (BMI) technology suffers from major issues such as long term stability, low information rate, and high electrode counts needed that has limited the translation of technology from research setting to clinical and household environment. In this study, we propose a novel method of extracting volitional signals from the descending tracts of the spinal cord dorsolateral column above the point of injury and utilizing them as control signals for a spinal cord-computer interface. This study proposes that accessing signals at the spinal level could resolve the stability and information rate issues and perhaps reduce the number of electrodes required due to the unique features of spinal cord neuroanatomy.

Several studies have confirmed that multiple brain areas are involved in any given behavior [11-19]. The prediction accuracy of a BMI depends not only on the number of neurons sampled from multiple cortical areas but also on the type of motor parameter being predicted [20]. Accurate prediction of limb kinematics becomes a challenge if only one cortical area is tapped. There is also a limit on the number of electrodes that can be implanted in a subject, which needs to be increased in order to expand the repertoire of a BMI to include a wide variety of tasks. Due to the nature of distributed coding principle [21] in the brain, the spinal cord may provide a favorable alternative [22] for tapping neural signals and using them as command signals.

Reports in the spinal cord injury literature indicate that the proximal sides of the motor fibers are still functional after an injury that severs the spinal cord. The distal portions of the axons go through “Wallerian” degeneration. However, the time course of degeneration for the proximal corticospinal tract (CST) is much slower and a significant portion of the fibers is preserved even years after injury [23-26]. Investigations of corticospinal axons have shown variable amounts of retrograde degeneration in several species after transection of the medullary pyramids [27-29]. Fishman [25] reported that spinal cord sections within a few spinal segments of the injury were grossly depleted of CST axons. However, the number of axons was close to normal at a distance rostral to the injury, regardless of the duration after the lesion. Clinical studies have also shown that tetraplegic patients were able to voluntarily modulate primary motor cortex (M1) spiking activity several years after injury [30,31]. All these reports suggest that descending motor signals may be accessible from the spinal cord segments above the point of injury.

The axons carrying these descending signals from different brain areas are tightly bundled together in the spinal cord tracts that innervate various regions of the body depending upon the cord level. The spinal cord contains two major descending systems: the lateral and the medial systems. The medial descending system is mainly involved in posture related activities by integrating vestibular, visual, and somatosensory information [32-34]. The lateral system, consisting of the corticospinal tract (CST) and the rubrospinal tract (RST), is involved in producing skilled forelimb movements and constitute the main pathways for motor control across species [35-52]. A single high-density microelectrode array (MEA) implanted in the cervical region would be able to record motor signals both from the CST and the RST in humans because the two tracks intermingle as they descend in the dorsolateral funiculus. In the rat, however, unlike the humans the major component of the CST travels separately from the RST in the ventral most portion of the dorsal funiculus of the spinal cord [43,53-55].

In this study, descending signals of the RST in the dorsolateral funiculus from the rat cervical cord were recorded to investigate the feasibility of using these signals as a means of generating command control. The main focus of this study was to demonstrate the functional characteristics, *i.e.* signal amplitudes, signal-to-noise ratio, and decoding characteristics of the neural signals recorded for periods lasting up to 3 months, and thereby presenting evidence for potential use of these signals in the context of brain-machine interfacing. Stability of signals was assessed based on mean signal amplitudes and signal-to-noise ratios. Forelimb kinematics such as hand position, elbow angle, and hand velocity were also reconstructed using the neural signals to demonstrate that the information encoded in these signals is correlated to the motor function.

## Methods

### Behavioral training

All procedures were approved by the Institutional Animal Care and Use Committee (IACUC), Rutgers University, Newark, NJ. Four adult (4–5 months old) male Long Evans rats, all weighing between 300–350 g were used for this study. All animals were trained to reach and grasp food pellets (Bio-Serv, NJ) through a small aperture prior to electrode implantation surgery. The dimensions of the clear plexi-glass training box were 20 x 20 x 20 cm. The aperture was a 3 x 3 cm opening in the box wall about 2.5 cm from the floor and the right-hand side wall. Food pellets were dropped one at a time by the experimenter during each trial. The animals were kept on a feed-restricted diet and then maintained at approximately 85% of their initial body weight during the study period. There was no restriction on their water intake at any time and they were fed normally during weekends. Animal training started approximately ten days after the beginning of food restriction. The animals were placed in the training box for 2–3 sessions for acclimatization after which food was presented as a reward. The animals were considered trained when they attained 90% success level in food reaching, grasping, and retrieval through the aperture, which usually required 8–10 training sessions each lasting approximately 30 minutes. All animals in this study were right-handed.

### Electrode

Custom-made 15-channel microelectrode arrays (MEA) (Blackrock Microsystems, Inc, UT) were used in this study. Electrode shanks were custom arranged in a 5 x 3 matrix and the total dimensions of the MEA were 2 x 1.2 mm. Each shank was 1 mm long, 80 μm thick at the base and tapered to a fine tip. The shank tips were coated by the manufacturer with platinum (approximately 50 μm) that served as the recording surface. Electrical connection to the headstage amplifier was made *via* a micro-connector (Omnetics, MN).

### Surgical procedure

**Figure 1:**
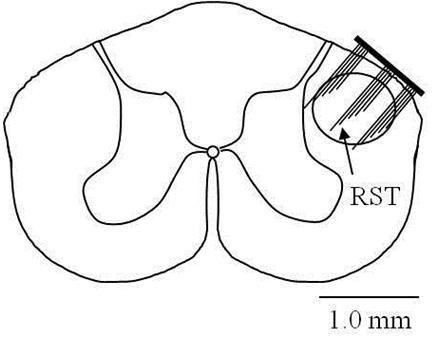
**Implant location:** The drawing depicts the location of the MEA implant in the dorsolateral funiculus (DLF) at the C5 level in the rat spinal cord. The circle depicts the location of the DLF. The DLF in rats primarily contains the rubrospinal tract. However, a similar location in primates and humans contains the corticospinal tract.

Animals were operated on after they were completely trained for the reach-to-grasp task. Deep anesthesia was induced using sodium pentobarbital (30 mg/kg, IP) and additional doses of 6 mg/kg (IP) were administered as needed to maintain it. Bupivacaine (0.2 ml, SC) was injected at the incision site for local anesthesia. Dexamethasone (0.2 mg/kg, IM) was used at the beginning of the surgery to prevent edema in the central nervous system and atropine (0.1 mL, IM) to improve cardiac function and reduce upper airway secretions. The animal’s head was fixed in a standard stereotaxic frame after induction of anesthesia. An incision was made along the midline over the cranium. The skull was exposed after retracting the skin and removing the periosteum using a scalpel. Six holes were drilled into the skull using a fine drill bit and metal screws (Plastics One Inc, VA) were screwed into the holes. Platinum wires wound around the screws served as the ground connection. The head-stage micro-connector was then fixed to the skull using dental acrylic. A second incision (about 4-5 cm) was made from the end of the micro-connector on the back of the neck down in the midline. Muscle layers were separated using blunt scissors to access the vertebra. Laminectomy was then performed at C5/C6 level to expose the spinal cord. The dura was punctured with a 27 gauge needle and then cut using microscissors. The cut dura was reflected over the sides. The MEA was slowly pushed free-handed into the right dorsolateral funiculus with the long side of the electrode bordering the dorsal entry zone (Figure 1) at C5 level under 40x microscopic vision. A thin layer of cyanoacrylate glue was applied on and around the MEA to attach it to the surrounding pia matter and seal the dural opening. The muscles were then sutured back in layers using 5.0 absorbable sutures. The wire bundle running from the MEA to the micro-connector was not tied to surrounding structures in order not to create points of high stress on the wires. Buprenorphine (0.05 mg/kg, IM) was given twice daily for 3 days post-operatively as an analgesic. Antibiotic ointment was applied if needed until the wounds healed completely.

### Recording procedure

**Figure 2:**
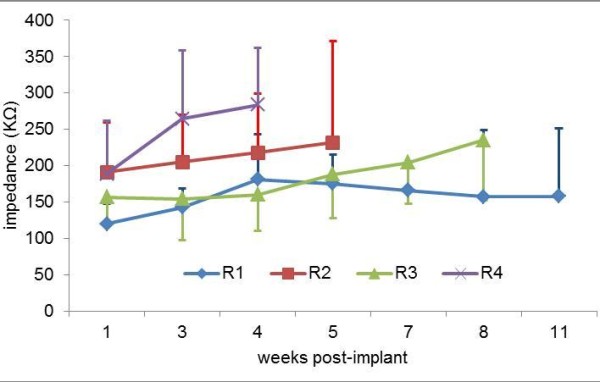
**Chronic electrode impedance:** Average impedances of all 15 electrodes in each week. R1-R4: Rat 1 through R4. The impedances were measured at 1KHz frequency and a constant current of 100 nA.

Recording sessions began approximately 2 weeks after the surgery. The recordings were performed approximately once a week, and each session consisted of 80–90 reaching trials. Animals were numbered as R1 through R4. R1, R2, R3, and R4 were implanted for 12, 5, 8, and 4 weeks, respectively. We were not able to record longer than 3 months primarily because of wire breakages or connector failures. Electrode impedances were measured (Electrode Impedance Tester, Model IMP-1, BAK Technologies Inc., MD) at 1 kHz frequency and 100 nA current prior to each recording session to keep track of changes over time and whether a channel needed to be dropped out of analysis because of electrode failure. Figure 2 shows the temporal variation of the weekly average impedance values from all electrodes in all animals during their recording period. In general, the impedance values ranged between 100-300KΩ and a gradual increase in average impedance was observed during the implant period.

The impedances were initially found to be low immediately and a few days after surgery but they returned to near *ex-vivo* values after 10–14 days and did not show a major change comparable to the initial drop for the rest of the study period. The initial instability in electrode impedances has been reported because of the acute tissue response due to stabbing injury caused by implantation [80-84]. As the tissue encapsulation begins to occur around the microelectrode tips by the second week, the implant becomes more stable and impedances return to their pre-implant values.

A 32-channel, 100-gain headstage amplifier (Triangle Biosystems, Inc) was used to interface the MEA with the data acquisition card (National Instruments PCI 6259) and the computer. A trial consisted of five seconds of neural data as well as video images acquired simultaneously using a Matlab program. Neural data and video images were time stamped using the functions in Matlab’s data and image acquisition toolboxes. Neural signals were acquired at a sampling rate of 30 kHz. Video frames were captured with a camcorder from the right side of the rat at 30 frames/s and then deinterlaced to form movies at 60 fields/s.

### Immunohistology

At the end of the study period, the animals were anesthetized and perfused with heparinized saline followed by 4% neutral buffered formaldehyde (NBF), freshly prepared from p-formaldehyde. The spinal cord segment harboring the electrode array was explanted and further fixed for 24 hours in the NBF and then rinsed in phosphate buffered saline, pH 7.4. The fixed cord segment was trimmed to size under stereo microscope and embedded in paraffin. For sectioning, the paraffin block was oriented such that coronal sections could be taken parallel to the MEA substrate. Five 6 micron serial sections were taken for staining at 20 micron intervals until the MEA shanks could be seen through the remaining paraffin block. Serial sections were stained with Luxol fast blue-cresyl violet (LFB) for evaluation of myelin and other cellular components of tissue reaction.

Immunostaining with anti-GFAP (glial fibrillary acidic protein) was used for evaluating astrocytic activation. The sections and the remaining block were deparaffinized and subjected to antigen retrieval using heat in a computer controlled pressure cooker (Pick Cell Laboratory Retriever) for immuno-staining. The tissue sections were rinsed with phosphate buffered saline (PBS) once. The sections were fixed with 100μL 4% paraformaldehyde for 20 minutes at room temperature followed by a rinse with PBS. The sections were then blocked using 100μL PBS containing 0.1% triton X-100, 10% normal goat serum (Invitrogen, PCN 5000), and 1% bovine serum albumin (BSA) (Fisher Scientific, BP671-1) at room temperature for 45 minutes. This was followed by rinsing the sections with 50μL 0.1% BSA in PBS for 10 minutes and incubating with 40μL PBS overnight at 4°C containing 1:25 rabbit-anti-GFAP (100 μg/mL) (Invitrogen 18–0063) as the primary antibody. The sections were then rinsed with 50μL 0.1% BSA in PBS for 10 minutes followed by incubating with 40μL PBS at room temperature for one hour containing 1:200 goat-anti-rabbit IgG (Alexa Fluor 488, Invitrogen A11008) as the secondary antibody. The sections were then rinsed with 50μL 0.1% BSA in PBS twice for 10 minutes each before putting the cover slip on the slides. Stained sections were evaluated by fluorescence microscopy or bright field microscopy as appropriate.

### Data analysis and results

#### Raw neural signals

Data analysis was performed using MATLAB (Mathworks, MA). Signals were recorded and referenced with respect to one of the shanks on the array and a Pt wire tied to the skull screw was connected to the data acquisition board ground. The reference electrode could alternatively be placed on the spinal cord surface. However, a differential recording with respect to this electrode would be contaminated with sensory signals from the spinocerebellar tract located near the surface. The configuration described above effectively removed the common-mode signals from distant sources such as skeletal muscles, the heart, and the brain.

**Figure 3:**
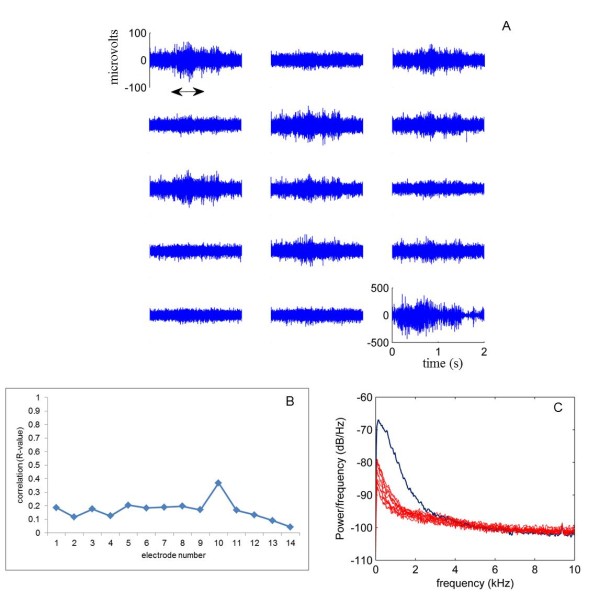
**Raw neural signals:A:** Two seconds of neural data from a representative behavioral trial band-pass filtered between 300-3000 Hz from R1. Arrow in the figure marks the duration of the reach-to-grasp task. Right bottom-most plot is the EMG signal recorded from a Pt wire inserted into the muscles near the MEA. **B:** Cross-correlation in the above trial between each one of the electrode channels and the EMG signal from the wire electrode to show the lack of similarity between the two kinds of signals. EMG contamination in the neural signal was low suggested by small R-values. **C:** Power spectral density of the signals calculated using the Welch periodogram method. Red traces are the 14-neural channels whereas the blue trace is for the EMG signal. The neural and muscle activities occupy different frequency bands with the amplitudes observable above the noise floor.

Raw signals were band-pass filtered between 300 Hz-3 kHz to extract multi-unit activity of the axons in the dorsolateral column. Neural activity increased above the noise floor each time the animals performed the reaching task as exemplified in Figure 3A where 14-channels of neural data is shown from a representative reach-to-grasp trial. Filtered neural signals had peak-to-peak amplitudes up to 100 μV. The behavioral onset could always be detected as an abrupt increase in the signal amplitude. Each of the electrodes in the MEA records unique activity patterns as the common-mode signal was removed by the referencing method. Activity of most electrodes were observed to be correlated with the behavior timing but some electrodes did not show any change in their baseline level during the behavior. A separate 50 μm Pt wire was inserted into the muscles next to the MEA implant site to record their activity and study their correlation to spinal signals. The EMG signal had peak-to-peak amplitudes up to 1 mV, about 10 times larger than the neural signals. Cross-correlation was performed between each of the electrode channels and the EMG signal for a trial (Figure 3B). In general, the EMG activity pattern was very different from the neural activity as indicated by the low correlations. This suggested that the neural channels did not suffer from EMG contamination from the neighboring muscles.

The difference in signal characteristics was further investigated by the power spectral density (PSD) of the raw neural and EMG signals during the representative trial (Figure 3V). The PSDs show significant power at the lower end of the spectrum (f < 200 Hz) and decrease by frequency. The power spectrum for EMG activity is significantly higher than that of the neural activity and contains frequency components above 2 kHz.

**Figure 4:**
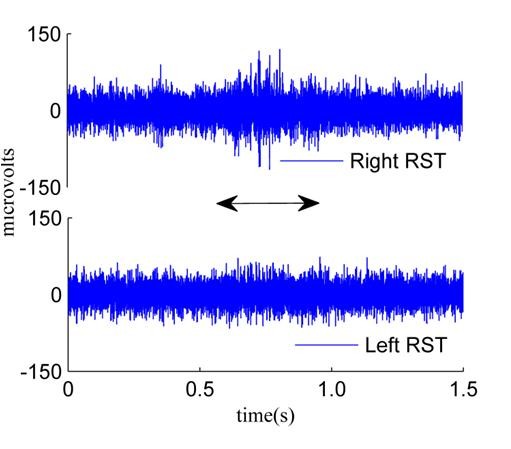
**Raw neural signals from bilateral implantation:** Bilateral implants using 25 μm diameter Pt/Ir wires in a separate study showing the difference in neural activity from the contralateral (left RST) and ipsilateral (right RST) spinal cord during a reach-to-grasp task. Signals recorded from the ipsilateral cord increase during the behavior while the contralateral cord activity does not.

We performed a similar study in a separate group of animals (n = 4) described in detail in [56] where four 25 μm diameter wires were implanted bilaterally into the dorsolateral column at the C5 level of the spinal cord (Figure 4). Neural activity was observed to increase above the baseline level from the ipsilateral cord as compared to signals from the contralateral cord that did not show any change in signal amplitude during the behavioral task. Signals recorded using individual microwires were similar in amplitude (~100-150 μV peak-to-peak) to that recorded from Utah MEAs of this study. Figure 4 provided further evidence that the signals recorded were indeed axonal potentials and not artifacts arising from different sources.

#### Signal amplitude stability

In order to quantify signal amplitude stability over time, signals were rectified and averaged within the ~500 ms window that the behavior occurred in each trial. Mean and standard deviations of the signal amplitudes were calculated across multiple trials and sessions. One-way analysis of variance (ANOVA) was then performed between the mean amplitudes of all trials within different sessions to investigate signal amplitude stability during the study period. Tukey’s post-hoc analysis was then used to determine the sessions where the mean amplitudes differed significantly.

**Figure 5:**
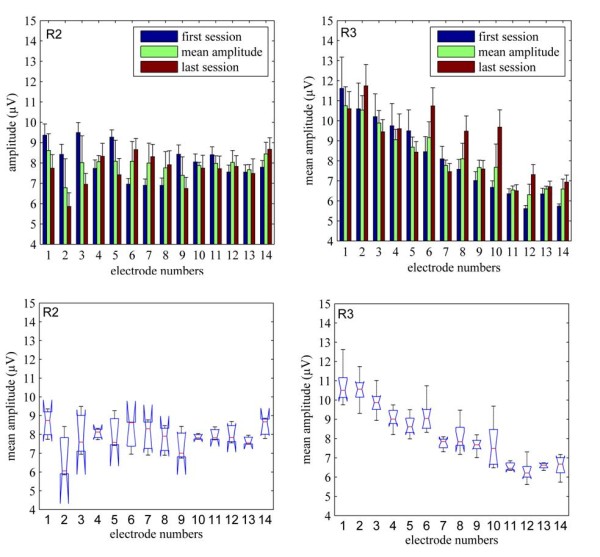
**Signal stability:** Mean signal amplitudes as measured from the rectified-averaged version of the data (top figures). The blue and brown bars are the means of trials from the first and last sessions to show an overall change in the mean signal amplitude level during the implant duration for the respective animal. The green bar in the middle shows an average of the mean amplitudes calculated for all the recording sessions. The box plot (bottom) shows the spread of the mean signal amplitudes from all sessions in R2 and R3 (blue cones) as well as the outliers (solid lines).

Figure 5 (top panel) shows the changes that occurred in the mean signal amplitudes from the first to the last recording sessions in two of the animals. The small standard deviations suggest that the signal amplitudes remained fairly stable during the recording period, which was also confirmed by the ANOVA analysis. The percentage of electrodes with stable mean signal amplitudes (p-value < 0.05) were 50% in R1, 100% in R2, 72% in R3, and 85% in R4. The box plots in Figure 5 (bottom panels) show the spread of mean amplitudes across all sessions, ranging between 6-12 μV. Note that these amplitudes were measured from filtered and rectified-averaged versions of the recordings and therefore they are much smaller than the raw signals.

#### Signal-to-noise ratio

**Figure 6:**
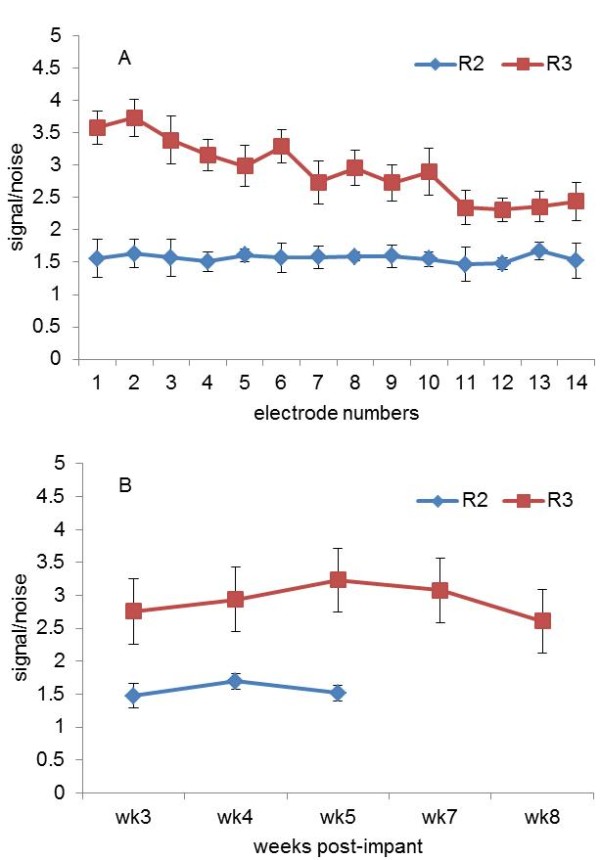
**Signal-to-noise ratio:A:** Average signal-to-noise ratios (SNR) for each electrode in R2 and R3 for their respective implant durations. **B:** Temporal variation of SNR averaged from all 14 electrodes and across sessions in each week.

Signal was defined as the standard deviation of the neural signal during the behavior. The baseline or the noise floor of a channel was defined as the standard deviation of the channel during the quiet phase of the reach trial when the animal was waiting for the food. Signal-to-noise ratio (SNR) was calculated as the ratio of these two values. Note that the baseline noise may contain steady neural activity unrelated to the motor behavior. Our definition of SNR considered non-phasic components of the signal as noise. Using standard deviation is a robust way of quantifying signals that are stochastic in nature. Multi-unit neural activity is a very random signal in terms of its amplitude distribution. Thus, using its first order moment within a time window is much more robust than taking the peak value for instance, or any other instantaneous value. The mean SNRs are plotted in Figure 6A for R2 and R3 across electrode contacts and against implantation time. The mean SNR was found to vary between 1.0-3.5. The SNR remained fairly stable during the recording period (in all animals) as suggested by the small standard deviations. One-way analysis of variance followed by Tukey’s post-hoc analysis suggested that 50% of the electrodes in R1, 100% of the electrodes in R2 and R3, and 70% of the electrodes in R4 had stable SNR during the recording period (p-value < 0.05). Figure 6B shows the temporal variation of SNR averaged for all 14 electrodes across all sessions in each week. There is a small decline in the SNR from R2 after the 4-week period, perhaps suggesting some scar tissue formation around the electrode tip.

#### Decoding characteristics of the signals

The rat’s shoulder, elbow, and hand were marked manually (using the black-white patterns on the animal’s fur as landmarks) in each video frame retrospectively to track the rat’s arm position during the behavioral task. Cosine formula was used to calculate the elbow angle from the distance measurements between the three markers. The relative hand position was calculated by subtracting the x- and y- coordinates of the elbow from that of the hand. The vectoral velocity of the hand was found with respect to the elbow by differentiating the hand position. Finally, rectified-averaged versions of the principal neural components were down sampled to match the sampling rate of the kinematic variables and used in reconstructing them.

**Figure 7:**
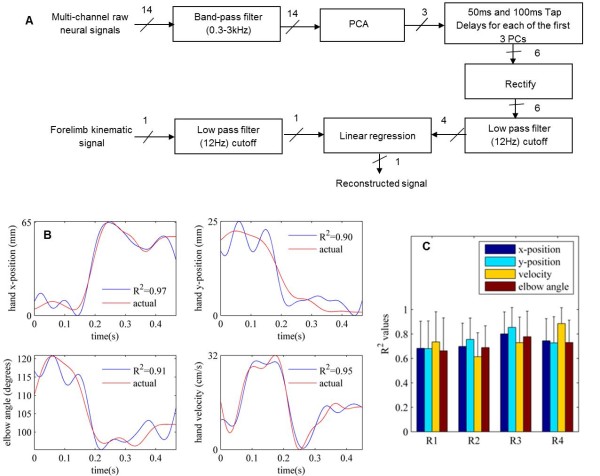
**Kinematic reconstruction: A:** Flow diagram for reconstruction of the forelimb kinematics from the neural signals. **B:** Reconstruction of forelimb kinematics (hand x- and y-positions, elbow angle, and hand velocity) during a representative reaching trial in R1. Traces in red are the actual kinematics measured from the video images and the blue traces are the reconstructed versions obtained by linear regression. The vertical axes are the actual distances (x- and y-positions) and the elbow angle calculated from the video images. Both neural and kinematic signals are low-pass filtered with a 4^th^ order Butterworth filter with a 12 Hz corner frequency. **C:** Average correlation **(**R^2^) values for the kinematic variables reconstructed from all trials in all four animals.

The flow diagram for the reconstruction of each forelimb kinematic from the neural signals is shown in Figure 7A. Principal component analysis (PCA) was applied on 14 channels of filtered neural signals. The first 3 PCs were taken into consideration for analysis as they mostly accounted for over 80% of variance in the data. In general, the first three PCs were sufficient for reconstructing the forelimb kinematics, thereby reducing the dimensionality of the data from 14 neural channels to 3 PCs. Tap-delayed versions, *i.e.* sets of differentially lagged copies with a fixed delay of 50 ms and 100 ms, of the first three PCs were generated to account for variable delays for the neural signals to travel from the spinal cord to the forelimb. For example, a 50 ms tap delayed version of the neural signal was generated by taking into consideration the signal that occurred 50 ms prior to the beginning of the behavior. Similarly, a 100 ms tap-signal referred to the neural signals that occurred 100 ms prior to the behavior. Therefore, creating a 50 ms and 100 ms tap-delayed version of each of 3 PCs resulted in 6 channels of information that was fed into the linear regression model (Equation 1). The tap-delayed versions of the PCs were then rectified and low pass filtered using a 4^th^ order low-pass Butterworth filter with a cut-off frequency of 12 Hz to extract the slow changing components. The same filter was also used for the forelimb kinematics extracted from video images as described above. The general linear regression model represents the relationship between a continuous response ***y*** (kinematic function) and a continuous predictor ***x*** (PCs) in the form:

(1) $$y=\beta_{0}+\sum_{i=1}^{6}\beta_{i}x_{i}+\varepsilon$$

The response was modeled as a linear combination of regressor variables (multiple linear regression model), plus a random error ϵ. The regressors in the model were the 50 ms and 100 ms tap delays of the first three principal components. The Matlab’s *regress* function computed the β coefficient estimates of the above model (1). The success of regression was evaluated by the coefficient of determination (R^2^ values) between the reconstructed and measured kinematic variables.

Measured and reconstructed kinematic variables are plotted in Figure 7B from a representative trial. In general, the lower frequency components were represented with higher fidelity. This analysis was extended to all animals. Figure 7C shows that the forelimb kinematics were successfully reconstructed from the spinal cord signals with correlation coefficients of R^2^ > 0.7 in all animals, which strongly suggest a causal relation between the spinal cord signals and the forelimb kinematics.

#### Immunohistology

**Figure 8:**
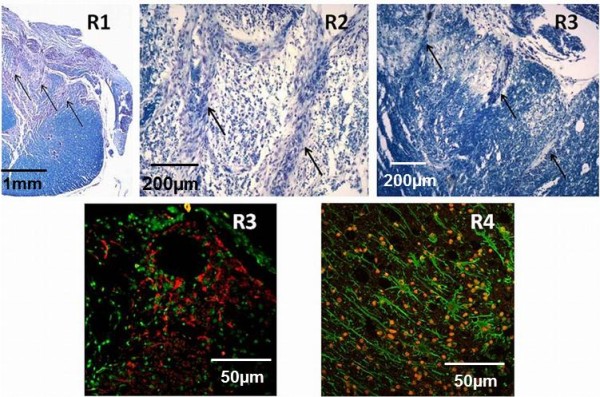
**Histology: Spinal cord section stained with Luxol fast blue (top pictures).** Arrows point to the electrode tracts separated by 400 μm. Bottom images obtained using fluorescence microscopy show an electrode tip in the coronal plane where the nuclei are stained in red and the astrocytes in green. The circular void in the bottom left image is made by the electrode tip. The bottom right image shows the astrocytes eliciting a reactive phenotype. This image was taken near the electrode tip

The pictures in Figure 8 are C5 sections stained for either Luxol Fast Blue-cresyl violet (top three pictures) or for astrocytes (bottom pictures). The arrows in the top image point the locations of electrode tips. The electrode shanks had some encapsulation around them (R1 and R2 after 2 months of implant and R3 after 3 months of implant). The substrate of the MEA evoked significant tissue response near the cord surface because of its size and rigidity in all the animals (visible in top left). An electrode with a flexible base that can conform to the cord shape would be better suited for spinal cord implants. The sections stained for GFAP expression did not show severe astrocytosis in any of the rats (bottom pictures). Rat R4 had the largest degree of astrocytosis among all animals. No quantitative analysis was done on the tissue response.

## Discussion

In this work, we investigated a novel approach for a human-machine interface where the activity of descending tracts in the spinal cord is proposed as a means of generating motor control signals. The main focus of this study was to show: 1) the feasibility of long-term recording from the spinal cord descending tracts, 2) that these signals can be recorded with similar fidelity without deterioration of the signal-to-noise ratio after the first few weeks of acute tissue response, and 3) show that the information encoded in these signals are correlated to the motor function. Rousche and Normann [57] have shown reliable recordings from the cat sensory cortex for several months using arrays. Recent studies reported that stable signals could be recorded over long term using these electrodes in non-human primates [58,59] and humans [9]. However, all of these investigations involved electrodes implanted in the cerebral cortex. This study proposes an alternative method for human-machine interfacing by shifting the paradigm from the brain to the spinal cord.

Whishaw [60] and Sacrey [61] have shown similarities in forelimb movements and velocity profiles between humans and rats, suggesting that the rat is a suitable model to study the neural control of forelimbs. The RST at the cervical level contains information required for controlling forelimb movements in rats and other species [35-41]. The same implant site in the dorsolateral funiculus of the human spinal cord would primarily record the neural activity of the lateral corticospinal tract (rather than the RST), which is the primary descending pathway in humans. Segments C5-C6 were chosen as the implant site for recording RST signals in rats since it has been reported that RST axons and its collaterals present in C5-T1 spinal cord segments terminate onto the motor neuron pools at these levels that are involved in the control of the forelimb movements [62]. Note that electrode implants at a higher level may have compromised diaphragm function leading to respiratory complications.

The dorsolateral funiculus occupies a large portion of the cervical spinal cord across species. In the rat, the RST tract is found in the dorsolateral funiculus just underneath a thin layer of sensory tract, *i.e.* dorsal spinocerebellar tract and thus accessible with electrodes of 1 mm length. A surface electrode would not be suitable as it would be recording mostly sensory information. Therefore, commercially available MEAs with penetrating shanks were used as the electrode of choice. The rat spinal cord is too small for implantation of a 100-channel standard Blackrock array. A custom-made 15-channel array with 1 mm electrode shanks was selected instead. Increasing the number of channels would have enabled us to sample a larger set of signals, but it would also increase the size of the MEA hence the trauma to the spinal cord.

Microelectrodes implanted into the white matter of the spinal cord are surrounded by a dense population of axons. Multi-unit activities recorded by these micro-electrodes may thus be insensitive to micro motions. This may be the most important factor for the stability of the recordings. The outcome measures to evaluate long-term stability in the recordings were the variability of the SNR and the mean amplitude of the signal during behavior. The mean signal amplitudes and SNRs were found to vary during the implant duration (Figure 5). However, the signals do not deteriorate substantially or disappear after the acute tissue response, or change in amplitude during the recording time, some of which are common problems with single neuron recordings from the cortex. Some signal amplitude change can also be attributed to the variations in the way the rats perform the behavior. The SNR values in general were low in our case, which may be due to the background neural activity that was considered as the baseline noise in our analysis. The signal mean for the duration of the entire behavior is a more robust parameter to quantify the activity rather than the signal peak, which fluctuates substantially from trial to trial. Using the standard deviations of signal and noise also produced modest yet robust values for SNR.

It is still debated whether the cortical area M1 encodes forelimb dynamics [63-69] or kinematics such as position, velocity, and acceleration [70-73]. Vargas-Irwin [74] showed that M1 encodes sufficient information to reconstruct some of the kinematic features such as joint angles of arm, wrist, and hand during a reach-and-grasp task. Miller and Sinkjaer [75] showed that the magnocellular portion of the red nucleus (RNm), where the RST originates [76] encodes the dynamics of limb muscle activity. The spinal cord circuitry is responsible for integrating these signals to activate various groups of muscles. Therefore, it is plausible that the signals in these spinal cord motor tracts encode both dynamic and kinematic parameters of a voluntary movement.

In this study, we concentrated only on forelimb kinematics. The forelimb motor function was hypothesized to have a stronger correlation to the spinal cord motor signals since these signals are closer to the target muscles in the signal pathway than the higher centers in the brain. The degree of success in reconstructing limb kinematics, determined by the correlation coefficient, was the outcome measure. High degrees of correlation were obtained for all the reconstructed kinematic functions in all the animals suggesting that some aspects of the forelimb kinematics are encoded by the spinal cord signals.

The performance of any neural prostheses would depend on the reproducibility of the control signals. The size of the implanted electrode, insertion technique, and the glial response [77-85] are some of the major factors that determine the stability of the interface over the long term. Recent reports by Bamford [86] showed that spinal cord tissue tolerated microwire implants up to one month post-implant. However, Utah arrays used here were significantly larger and rigid in structure compared to the single microwires. Despite the evoked immune response and the tissue encapsulation observed around the electrode shanks, animals used in the study were not at any time during the recording period paralyzed due to the implant. Paralysis was observed in some cases where the spinal cord was damaged during the surgery. Those animals were euthanized and no data were included in the analysis from them. The immune response also did not significantly degrade the neural signals during our recording period as shown by moderate changes in the mean signal amplitude between the first and the last recording sessions (Figure 5).

### Limitations of the study

One of the major limitations of the study so far has been the inability to record for more than three months. This is mainly due to the lack of a suitable electrode for the rat spinal cord. The use of surface electrodes is attractive because of reduced invasiveness compared to the penetrating electrodes but the anatomical locations of the spinal cord motor tracts does not allow us to use these electrodes. The signals recorded from a surface electrode would contain sensory contamination from the spinocerebellar tracts residing close to the spinal cord surface on the dorsolateral side. Penetrating Utah MEAs were used for study since they were commercially available and the electrode shanks were arranged in well-defined positions. There was also not a large variation between electrode sizes and impedances due the fabrication process. These MEAs were able to record stable signals in our study up to 3 months. However, histological studies showed that they caused significant damage around the implant site. These MEAs also had a solid and thick base which contorted the shape of the cord as seen in histological pictures.

We tested custom-made 25 μm diameter Pt-Ir microwire electrodes implanted bilaterally in a separate study [56]. Using these electrodes, we were able to show that the contralateral cord did not contain observable activity during a task (Figure 4). These microwires were individually inserted into the cord and the surgical procedure was significantly more tedious and longer than MEA implants. However, the histological analysis in these animals showed that the glial scarring and the tissue response were significantly lesser compared to the MEA implants. Only mild astrocytosis and gliosis were observed in the microwire implants. We have not been able to record for more than four weeks in these microwire implanted animals because of wire breakage.

Another major limitation of this study was that the reaching kinematics was reconstructed within individual trials and not generalizable across a set of test trials using the beta coefficients from a training set. The variation in beta coefficients might be because of the slight differences in how the animal reaches for the food pellet between trials. Coefficient variations may have also been due to the use of a single camera to capture the forelimb kinematics in one plane only. Our laboratory has started to use a three camera system with faster frame grabbing rate to improve the behavioral imaging.

## Conclusion

Despite the limitations of this study, the results shown here can have major implications in the field of brain-machine interfacing by providing an alternative target site for accessing control signals. In this study, we have demonstrated the feasibility of chronically recording spinal cord descending signals in the motor tracts. We have also shown that forelimb parameters could be successfully reconstructed from spinal cord signals suggesting the information encoded in the spinal cord descending pathways can be utilized as an alternate implant site for tapping into volitional signals. Multi-unit recording method in general may provide more robust signals than the single neuron recordings as commonly used in the central nervous system.

## **Competing interests**

The author(s) declare that they have no competing interests.

## **Authors’ contributions**

AP and MS conceived and designed the experiments. AP performed the experiments and analyzed the data. MS contributed materials/analysis tools. AP and MS wrote the paper. Both authors read and approved the final manuscript.
